# Supplementary material for: Development of Antimicrobial Stewardship Programmes in Low and Middle-Income Countries: A Mixed-Methods Study in Nigerian Hospitals
Source: Antibiotics (Basel). 2020 Apr 23;9(4):204. doi: 10.3390/antibiotics9040204 (PMC7235739; doi:10.3390/antibiotics9040204)
Supplement: Supplementary file 1 [file antibiotics-09-00204-s001.pdf]

Supplemental Materials

Supplemental file 1: Data collection form
Supplemental file 2: Participants information and invitation sheets
Supplemental file 3: Consent forms
Supplemental file 4: Ethical approval letters

Supplemental file 1: Data collection form

DATA COLLECTION FORM- PATIENTS CASE NOTES				
**Title: A survey of antibiotic use and prescribing practices in the Niger Delta region of Nigeria**				
**Form No:**			**Date:**	
Hospital:	Level of Care:		Ward/Unit:	
Patient sex: M ☐ F ☐	Age:	Admitted: Y ☐ N ☐	No. of days: if yes	
Name of Antibiotic:	Class:		Route:	
Dose:	Duration:		Indication:	
**S/no**	**Outcomes**	**Yes**	**No**	**Any additional comments**
1	Generic prescribing?			
2	Is the prescribed antibiotic present in the essential drug list?			
3	Antibiotic prescription based on lab culture results?			
4	Any Potential interaction with other prescribed medicines?			
5	Appropriate prescription based on STG? Drug, dose, route, duration?			
6	Prescribed antibiotic available in the hospital?			
7	Was the prescribed antibiotic dispensed?			
8	Reason for prescribing: Empirical ☐ Prophylaxis ☐ Therapeutic ☐			
9	Total number of drugs prescribed:			
10	Estimated cost of antibiotic therapy in NGN:			
11	Prescriber: Doctor ☐ Pharmacist ☐ Nurse ☐ Others ☐			
**Form completed by:**				
**Date:**			**Signature:**	

Supplemental File 2: Participants information and invitation sheets

Information/Invitation Sheet

Title of Project: A survey of antibiotic use and prescribing patterns in Niger Delta Region of Nigeria and developing strategies to improve prescribing.

Name of Researcher:
Contact:

Email:
Phone:

Invitation

I would like to invite you to take part in an interview about antibiotics use and prescribing patterns in your hospital. Doctors are the main prescribers of medicines in the hospital; hence you are being invited to participate. Before you decide if you want to be interviewed, I would like to provide you with some information.

Background to this study

Antibiotic resistance is a global problem of increasing importance. Studies from developing countries have reported high and inappropriate use of antibiotics, a common cause of antibiotic resistance (As a result, conservation of available antibiotics is now essential to prevent the return of the post-antibiotic era, where common infections which had been treatable for decades can once again kill. There is therefore need for an urgent and coordinated action by the stakeholders in antibiotics use.

There is still limited data available on the antibiotic prescribing patterns in this area and also the opinions and the experiences of prescribers is very key in deciding any effective intervention. Up till now this has also not been fully explored in this region. Hence the design and execution of this study.

Aim of the research

This study aims to assess the antibiotic prescribing patterns in Bayelsa state of Nigeria. To evaluate the knowledge, attitude and perceptions of prescribers on prescribing of antibiotics. To identify and design an intervention(s) that will work in this setting to help improve antibiotics use. There are studies from other countries and regions, but suggestions may not be applicable to Nigeria and this region due to differences in practice settings.

Why have you been selected?

There is need to speak to diverse group of prescribers and policy makers. This is to obtain all perspectives on the use of antibiotics, your knowledge and expertise is very vital in obtaining the information needed for this study. After consenting, interviews will be arranged and conducted face to face.

Study type and procedures
It will be a one-off interview that will last for no more than one hour at a time and place that is convenient for you. I will ask questions on antibiotics use in the hospital, current practices, your experience and thoughts on what should and could be changed. I will be the only person with you during the interview, unless you prefer to have someone else with you. I will take some notes during the interviews, and also audio-record so that I do not miss anything you say. If you agree, this will speed up the interview and once I have transcribed the information which is anonymized, I will delete the recording.

Risks and benefits
There will be no risk to you if you decide to take part, but it may help us improve antibiotics use. If you do not wish to answer any question, it is totally fine to skip the question. You can also discontinue the interview at any point if there is any need to do so.

Anonymity and Confidentiality
I will keep everything you say confidential by not writing your name on my notes, storing the notes and tape recording securely. If the study team reports your opinions or ideas, your name will not appear and we will make sure that you cannot be identified. During the interview, I may call your name but when your interview is written up, I will give you a code number as opposed to your name so that you cannot be traced.

Voluntary nature of the study
Taking part in the study is voluntary. You do not have to give a reason to refuse to take part or to stop the interview. Refusing to participate will not cause anything bad to happen. We do not pay for being interviewed but will give out a little thank you pack.

Any Further Questions
Please feel free to contact me by email, phone or post if you have any further questions. (Contact details have been provided at the beginning of this document).

All data will be collected and stored in accordance with the Data Protection Act 1998.
Thank you for reading this information sheet and for considering to taking part in this research.

Supplemental file 3: Consent forms

LONDON'S GLOBAL UNIVERSITY

UCL

Informed Consent Form

Please complete this form after you have read the Information Sheet and/or listened to an explanation about the research.

Project Title: **Antibiotic use and prescribing patterns in Niger Delta Region of Nigeria**
Researcher: **Eneyi Edith Kpokiri**

Thank you for your interest in taking part in this research.

If you have any questions arising from the Information Sheet or explanation already given to you, please ask the researcher before you sign this consent form. You will be given a copy of this Consent Form to keep and refer to at any time.

Participant's Statement

I agree that:

- I have read the notes written above and the Information sheet and understand what the study involves.
- I understand that if I decide at any time that I no longer wish to take part in this project, I can notify the researchers involved and withdraw immediately.
- I understand that my participation will be taped/video recorded and I consent to use of this material as part of the project.
- I understand that such information will be treated as strictly confidential and handled in accordance with the provisions of the Data Protection Act 1998.
- I agree that the research project named above has been explained to me to my satisfaction and I agree to take part in this study.

Signature………………………… Date………………………………

Supplemental file 4: Ethical approval letters

BAYELSA STATE HOSPITALS MANAGEMENT BOARD

P.M.B. 41, YENAGOA, BAYELSA STATE.

BAYELSA STATE HOSPITALS MANAGEMENT BOARD — HEALTH FOR SERVICE

Your Ref: ____________________

Our Ref: BSHMB/ADM/426/VOL.I/104

Date: 3rd February, 2016

Eneyi Kpokiri
Department of Practice & Policy
University College London
School Of Pharmacy
29-39 Brunswick Square
London, WCIN 1 AX
United Kingdom.

APPROVAL TO CONDUCT A STUDY ON ANTIBIOTIC USE AND PRESCRIBING PATTERNS

I am directed to refer to the above subject matter and to inform you that approval has been given to your application to **Conduct a Study on Antibiotic use and Prescribing Patterns.**

I am further directed to inform you that you should indicate the facilities you intend to use for the study.

Thanks for your usual cooperation, please.

Kalaowei Okponipere
For: Chief Medical Director

FEDERAL MEDICAL CENTRE
P.M.B. 502
YENAGOA

OFFICE OF THE HEAD OF CLINICAL SERVICES

INTERNAL MEMO

To	HOD – Pharmacy Department
From	Head of Clinical Services.
Date	16th September, 2015.
Subject	Re: A survey of antibiotic use and prescribing patterns in Niger Delta Region of Nigeria, and developing strategies to reduce antibiotic resistance.

The bearer, **Kpokiri Eneyi Edith** is a PhD student of School of Pharmacy, University College London. She has been granted approval to carry out a research work on the topic stated above.

Kindly assist her with the necessary data for her research work.

Thank you.

DR. P. P. F. NUMBERE
Head of Clinical Services.

RESEARCH AND ETHICS COMMITTEE

NIGER DELTA UNIVERSITY TEACHING HOSPITAL, OKOLOBIRI

CLEARANCE CERTIFICATE

Application form number: **NDUTH/ REC/ 0007/ 2015**.

Project Title: ***A Survey of Antibiotic use and Prescribing Patterns in Niger Delta region of Nigeria and Developing Strategies to reduce Antibiotics Resistance.***

Investigators: **Kpokiri Eneyi Edith**.

Department/Institution: **Practice & Policy, University College London.**

Date considered: **9th September, 2015.**

Decision of the committee**: Approved**.

Chairman: Professor Olu Osinowo Signature & Date..

DECLARATION BY INVESTIGATOR(S)

Protocol number:

To be completed in duplicate, and one copy returned to the Secretary, Research and Ethics Committee, Niger Delta University Teaching Hospital, Okolobiri, Bayelsa State.

I/we fully understand the conditions under which I am/we are authorised to conduct the above-mentioned research and I/we guarantee that I/we will ensure compliance with these conditions. Should any departure be contemplated from the research procedure as approved, I/we undertake to resubmit the protocol to the Research and Ethics Committee.

Signature .... Date....09/09/15....
